# Spontaneous Uterine Perforation due to Pyometra Presenting as Acute Abdomen

**DOI:** 10.1155/IDOG/2006/60276

**Published:** 2006-05-28

**Authors:** Loabat Geranpayeh, Mohsen Fadaei-Araghi, Behnam Shakiba

**Affiliations:** ^1^Department of Surgery, Sina Hospital, Tehran University of Medical Sciences, 14155-6537 Tehran, Iran^1^; ^2^Students' Scientific Research Center, Tehran University of Medical Sciences, 14155-6537 Tehran, Iran

## Abstract

Spontaneous perforation of the uterus is rare, its incidence being about 0.01% − 0.05%. We report a rare case of diffuse peritonitis caused by spontaneously perforated pyometra. A 63-year-old woman with severe abdominal pain was admitted to our hospital. Laparotomy was performed because of the suspicion of gastrointestinal perforation with generalized peritonitis. At laparotomy, about 900 mL of pus was found in the peritoneal cavity. There were no abnormal findings in the alimentary tract, liver, or gallbladder. A total abdominal hysterectomy with bilateral salpingo-oophorectomy was performed. Pathological investigation of the surgical specimen revealed endometritis and myometritis of the uterus; but there was no evidence of malignancy, and the cervical canal was patent. Although spontaneously perforated pyometra is rare, a perforated pyometra should therefore also be considered when elderly women present with acute abdominal pain.

## INTRODUCTION

An uncommon condition known as pyometra occurs when the natural drainage of the uterine cavity is comprised and pus accumulates within the cavity [1]. It has an incidence of 0.1%–0.5% [2, 3]. A spontaneous perforation of pyometra and subsequent diffuse peritonitis is rare [4]. Based on our knowledge, only 25 cases have been reported in English literature.

We report a patient that was admitted to our hospital for diffuse peritonitis caused by spontaneously perforated pyometra who was successfully treated by surgery.

## CASE REPORT

A 63-year-old Iranian woman was admitted to our hospital in December 2005 with the chief complaint of abdominal pain, vomiting, and fever that had developed one day earlier. She was a diabetic patient and she had no history of sexually transmitted disease. She had 3 pregnancies and all of her deliveries were normal vaginal deliveries. On physical examination, she looked acutely ill: her body temperature was 38.1°C, pulse rate was 126 beats/min, and blood pressure was 140/70 mmHg. Her abdomen was soft and was not distended. General tenderness with muscular guarding was recognized.

The results of laboratory studies on admission were as follows: white blood cell count: 18700/mL; red blood cell count: 3.4 × 106 /mL; hemoglobin: 10 g/dL; albumin: 2.4 mg/dL. Ultrasonography demonstrated a large amount of ascites in Morison pouch. Computed tomography (CT) with an intravenous injection of contrast material visualized a calcified uterine body and fluid and ascites without free air. She was diagnosed as having diffuse peritonitis due to a perforated gastrointestinal tract. After obtaining adequate informed consent, laparotomy was performed on the basis of a diagnosis of perforation of the gastrointestinal tract but revealed a perforated pyometra. At laparotomy, about 900 mL of pus was found in the peritoneal cavity. There were no abnormal findings in the alimentary tract, liver, or gallbladder. A total hysterectomy and bilateral salpingo-oophorectomy were carried out. A culture of the pus was negative. A histological examination of the resected specimen revealed pyometra. There was no evidence of malignancy. Postoperatively, intensive antibiotic therapy was performed. She recovered well and was discharged on the 15th postoperative day.

## DISCUSSION

Pyometra is common in postmenopausal women, and more than 50% of all patients of nonruptured pyometra are asymptomatic [7]. The incidence of pyometra becomes much higher with age and decline in activity; incontinence is also a significant risk factor [6]. Postmenopausal bleeding, vaginal discharge, uterine enlargement, and cramping pain are said to be the classic symptoms of pyometra, only bleeding and discharge were commonly reported in these cases [1] but this patient had no vaginal bleeding or discharge.

A spontaneous perforation of pyometra leading to diffuse peritonitis only rarely occurs. Once ruptured, the symptoms become severe and acute abdomen often develops; it is therefore important to make a differential diagnosis from other causes of acute surgical abdomens [2].

In conclusion, the diagnosis of spontaneous perforation of pyometra is rarely made preoperatively and the possibility of a perforated pyometra should therefore be considered when elderly women suffer from acute abdominal pain [5]. Their management is often difficult, and hysterectomy and bilateral salpingo-oophorectomy may be the best choice of procedure in these patients.
